# Repeatability and Reliability of the Rheumatoid Arthritis Foot Disease Activity Index in Spanish Patients: A Transcultural Adaptation

**DOI:** 10.3390/biology11010030

**Published:** 2021-12-26

**Authors:** Eva María Martínez-Jiménez, Héctor Pereiro-Buceta, Patricia Palomo-López, Emmanuel Navarro-Flores, Ana María Jiménez-Cebrián, Marta Elena Losa-Iglesias, Ricardo Becerro-De-Bengoa-Vallejo, Daniel López-López

**Affiliations:** 1 Facultad de Enfermería, Fisioterapia y Podología, Universidad Complutense de Madrid, 28040 Madrid, Spain; evamam03@ucm.es (E.M.M.-J.); ribebeva@ucm.es (R.B.-D.-B.-V.); 2 Research, Health and Podiatry Group, Department of Health Sciences, Faculty of Nursing and Podiatry, Universidade da Coruña, 15403 Ferrol, Spain; hector.pereiro@udc.es (H.P.-B.); daniel.lopez.lopez@udc.es (D.L.-L.); 3 University Center of Plasencia, Universidad de Extremadura, 10600 Plasencia, Spain; patibiom@unex.es; 4 Frailty Research Organizaded Group (FROG), Faculty of Nursing and Podiatry, Department of Nursing, University of Valencia, 46010 Valencia, Spain; 5 Department Nursing and Podiatry, Faculty of Health Sciences, University of Málaga, 29071 Málaga, Spain; amjimenezc@uma.es; 6 Faculty of Health Sciences, Universidad Rey Juan Carlos, 28670 Madrid, Spain; marta.losa@urjc.es

**Keywords:** questionnaire, arthritis chronic pain, pain measurement, foot

## Abstract

**Simple Summary:**

The Rheumatoid Arthritis Foot Disease Activity Index (RADAI-F5) is the first questionnaire designed to check the level of involvement in the feet of patients with rheumatoid arthritis. It is a validated and reliable five-question questionnaire in English. It is also considered suitable for clinical use. We aim was to translate and validate the Spanish version. The findings of this research suggest that is a valid, strong and trustworthy clinimetric tool that appropriately applies to the Spanish community and can be managed as a whole or in terms of its respective dimensions, such as arthritis activity in the foot, joint tenderness and swelling, and foot arthritis pain sub-scales.

**Abstract:**

**Background:** The Rheumatoid Arthritis Foot Disease Activity Index (RADAI-F5) questionnaire, based on five questions, is used to assess the severity of rheumatoid arthritis disease in the foot. Nowadays, RADAI-F5 has been validated in different languages; however a Spanish version was lacking. Therefore, the purpose of this research was to translate and validate the Spanish version (RADAI-F5-es). **Methods:** A cross-cultural translation of the RADAI-F5 questionnaire was performed from English to Spanish. To validate its use, 50 subjects with rheumatoid arthritis who responded to the translated questionnaire two times in an interval of less than 3 months were selected in order to verify the psychometric properties. **Results:** Excellent agreement between the two versions according to the Cronbach’s α was shown. Five domains with regards to arthritis activity in foot joint tenderness and swelling, foot arthritis pain, general foot health and joint stiffness were added together to obtain the total score. Excellent retest reliability was shown for the total score. Test/retest reliability was excellent for joint stiffness on awakening and foot arthritis pain domains. There were no significant differences among any domains (*p* > 0.05). There were no statistically significant differences (*p* = 0.000) for the mean ± standard deviations (SD) between pre- and post-tests (98.09 ± 15.42) [93.75–102.43] and 97.96 ± 13.88 [94.5–101.86] points, respectively). Bland–Altman plots or clinically pertinent variations were not statistically significantly different. **Conclusions:** The RADAI-F5-es is considered a valid and strong tool with adequate repeatability in the Spanish community.

## 1. Introduction

About 60% of subjects with rheumatoid arthritis have difficulty walking [1]. Additionally, in patients with rheumatoid arthritis, there is a high prevalence—estimated at 90%—of foot problems [1], among which pain and deformities often stand out, making it difficult to carry out daily activities, as well as posing difficulties in finding suitable and healthy footwear [2]. Patients with rheumatoid arthritis also have alterations in the ankle joint and difficulties in foot self-care due to the involvement of the disease in the hands and on the feet [2,3,4,5].

For early rheumatoid arthritis, different clinical practice guidelines recommend splints, orthoses and plantar orthoses for pain management [6]. In the stabilized rheumatoid arthritis phase, it is recommended that patients receive care from a multidisciplinary team that includes a podiatrist [7,8], with interventions aimed at preserving the joints with splints, orthoses and plantar orthoses, radiographic control at the beginning for the diagnosis, and subsequent annual follow-up exams to assess the extent of the disease [8,9,10,11].

It has been proven that foot involvement and the inability to walk are related to a negative impact on the quality of life of these patients [12,13]. The Rheumatoid Arthritis Foot Disease Activity Index (RADAI-F5) is the first questionnaire designed to check the level of involvement in the feet of patients with rheumatoid arthritis. It is a validated and reliable five-question questionnaire in English. It is also considered suitable for clinical use. The RADAI-F5 has been shown in studies to be able to measure the activity level of rheumatoid arthritis in the foot with greater capacity than the global disease activity indexes such as the 28 joint count disease activity score (DAS28-ESR) [3]. In addition, the RADAI-F5 allows evaluating patients for early and established rheumatoid arthritis. The psychometric properties of the RADAI-F5 questionnaire comply with the criteria of the International Society for Quality of Life Research (ISOQOL) [14] and Consensus-Based Standards for the Selection of Health Measurement Instruments (COSMIN) [15] as being a validated, repeatable questionnaire with the capacity for interpretation and response.

As RADAI-F5 aims to observe the involvement of rheumatoid arthritis locally in the foot, it is recommended as a complement to compound indices of disease activity, such as specific disability questionnaires including PROMS, or in the case of FIS rehabilitation, the latter with the ability to distinguish between inflammatory or mechanical-functional foot diseases, which is essential in early rheumatoid arthritis [16].

In research, cross-cultural validation, acceptability and trust are considered necessary and must be carried out in a protocolized way to allow the questionnaires to obtain the same properties [17,18].

For this reason, this research aimed to adapt the RADAI-F5 questionnaire from English to Spanish language in Spain, assessing its validity and reliability.

Our hypothesis was that the (RADAI-F5-es) would be a reliable clinimetric tool for the Spanish population.

## 2. Materials and Methods

A descriptive cross-sectional trial was designed and carried out from January 2021 to April 2021, fulfilling all criteria of the Strengthening the Reporting of Observational Studies in Epidemiology (STROBE) guidelines [19]. An ethics committee approved the study, and all procedures respected the ethical standards for human experimentation set out in the Declaration of Helsinki. Additionally, ethical approval was obtained from the Committee of the University of Extremadura—Code. 8/2021.

### 2.1. Sample Size Calculation

To calculate the sample size, G*Power 3.1.9.2 software (Heinrich-Heine-Universität Düsseldorf; Düsseldorf, Germany) was used to test correlation between two paired means regarding correspondence with a Spearman correlation coefficient of 0.40 and a 95% confidence interval (CI) for a two-tailed test, an error α of 0.05, a desired analysis power of 80% (error β = 20%), and the need for a final sample size of 46 participants [18].

The sample heterogeneity was tested with this tool for numerous and diverse foot statuses [18].

### 2.2. Procedure

The translation and validation process were developed using the RADAI-F5 as a clinimetric instrument [3].

First, a translation of the RADAI-F5 questionnaire was carried out. Second, patients with rheumatoid arthritis were selected through associations of patients with rheumatoid arthritis in Spain. Third, all subjects voluntarily accepted participation in the study, and before it started, were informed of the procedure and signed informed consent. The objective of translation and validation of the RADAI-F5 questionnaire was always to develop it as a clinical instrument [17].

The study research data were collected using a self-administered clinimetric tool. Sociodemographic characteristics (including sex, weight, height, and age) were entered after measurement of body mass index (BMI) using Quetelet’s equation [14].

Moreover, using images, patient foot exams were performed to evaluate the following: (1) metatarsal foot pain, (2) heel foot pain, (3) condition or deformities of the toes, (4) presence and types of arches, (5) foot morphology, and (6) skin pathology.

Then, patients completed the RADAI F5-es, a foot health questionnaire specifically designed for measuring foot arthritis alterations [3,20]. RADAI F5-es is composed of five domains that respectively concern arthritis activity in the foot, joint tenderness and swelling, foot arthritis pain, general foot health, and joint stiffness on awakening. Given adequate criteria, the results can be used to construct validity (Cronbach α = 0.90) and high retest scores (intraclass correlation coefficient = 0.80–0.91) [3]. The questions in the questionnaire can be answered using a visual analogue scale (VAS). With regards to score, zero points correspond to the worst state of health for the foot and a ten is the best possible condition.

### 2.3. Translation Procedure

First, the original authors of the RADAI-F5 clinimetric tool were contacted to request their permission for translation. The translation procedure was based on the worldwide guide [21,22]. The recommended methods for translation of the questionnaire—called forward and backward translation—were used to verify the intercultural correlations and validity of the questionnaire from England to Spain [18,21,22,23,24,25].

1. A. The authors of the original RADAI-F5 clinimetric tool were contacted to request their permission for its translation [16].
2. B. Two neutral polyglot Spanish interpreters performed the translation.
3. C. The version of the agreement in the following translations was developed by each translator individually.
4. D. Three of the authors of this investigation (ENF, MLLI and RBBV), all of them podiatrists, translated the ordered RADAI-F5 into Spanish and translated forward.
5. E. The comparison of the final version with the main statement verified conceptual concordance and whether interpretations were already translated, confused or discrepant.
6. F. An arrangement was made by the research team (4 podiatrists ENF, DLL, PPL, 2 nurses RBBV; MELI and 1 physiotherapists EMMJ) to finish the translation.
7. G. An intellectual evaluation for podiatric medical clinics was developed to provide feasibility and prevent potential misunderstandings [22].

### 2.4. Statistical Analysis

First, the normality of the distribution of the variables was verified with the Shapiro–Wilk test. The variables that obtained a *p* value > 0.05 were considered normal distribution. The independent student t test for parametric data and Mann–Whitney U test for nonparametric data were used to test differences between groups. In addition, the paired t test or Wilcoxon signed-rank test was used for parametric and nonparametric data, respectively, for the purpose of testing systematic differences between test and re-test.

With regard to each dimension, its score and total score, intraclass correlation coefficients [ICC] and the Cronbach’s alpha were analyzed. This parameter was employed to summarize the internal correlations of all items on a scale. Cronbach’s alpha was used to delineate the internal consistency of complete questions in one dimension. To clarify, a higher coefficient (ranging between 0.0 and 1.0) was considered more uniform for the domain with an excellent possibility of considering an individual support variable in the questionnaire.

Correlations were calculated for all items with the overall score. In addition, we tested whether Cronbach’s was improved by removal of any item. We calculated correlations of all items with the overall score using non-parametric Spearman test or parametric Pearson test.

Independent student t-tests were examined to find whether differences were statistically significant when showing a normal distribution. Considering the total score and each domain, reliability and internal consistency were analyzed through intraclass correlation coefficient (ICC) and the Cronbach alpha (α) with a 95% confidence interval (95% CI), respectively.

Moreover, Bland–Altman plots were calculated to check agreement and heteroscedasticity [26].

Relative to each domain score and total score, correlation and reliability and internal consistency were calculated employing Spearman (*r*_s_), intraclass correlation coefficients (ICC) and the Cronbach’s alpha, respectively. Cronbach’s alpha was employed to outline the internal consistency of whole questions on a dimension. Correlations of all items were checked with the equality degree. Moreover, if Cronbach’s alpha was removed, correlations of all items were checked with the overall degree using the nonparametric Spearman test.

Internal consistency was assessed with Cronbach’s alpha. Internal consistency above 0.7 was acceptable.

## 3. Results

All variables tested showed a non-normal distribution (*p* < 0.05), with the exception of weight and body mass index (BMI), which showed a normal distribution (*p* > 0.05).

The sociodemographic data are listed in Table 1.

The total data and all dimensions calculated during the test and retest showed a non-normal distribution (*p* < 0.05); therefore, the distribution was analyzed employing the nonparametric paired Wilcoxon signed-rank test in order to test systematic differences between the test and retest in Table 2.

### 3.1. Translation

The RADAI-F5 adaptation was developed with a very high degree of concordance between both translations; only small differences were found. The back translations between RADAI-F5 and RADAI-F5-es are in most of their questions identical. Intellectual evaluation verified excellent understanding and performance.

### 3.2. Test-Retest Analyses

Test-retest reliability results and systematic differences of the RADAI-F5-es instrument by subscales and total scores are indicated in Table 2 and Table 3. An adequate Cronbach’s alpha was indicated for the five domains of arthritis activity in foot [α = 0.965], joint tenderness and swelling [α = 0.965], foot arthritis pain [α = 0.975], general foot health subscales [α = 0.934], and joint stiffness on awakening [α = 0.987], as well as for the total marks [α = 0.980]. Excellent test re-test reliability (ICC [95%]) was shown for the total score (ICC = 0.980 [0.964–0.988]), and each subscale, such as activity in foot (ICC = 0.930 [0.876–0.960]), joint tenderness and swelling toms (ICC = 0.965 [0.938–0.980]) and foot arthritis pain (ICC = [0.956–0.986]) sub-scales. The Spearman’s correlations (*r*_s_) between test-retest were adequate for arthritis activity in foot (*r* = 0.858), joint tenderness and swelling (*r* = 0.914), foot arthritis pain (*r* = 0.965), general foot health (*r* = 00.865), joint stiffness on awakening (*r* = 0.935) subscales and total (*r* = 0.933).

No systematic differences were observed for dimension and total (*p* > 0.05).

**Figure 1:**
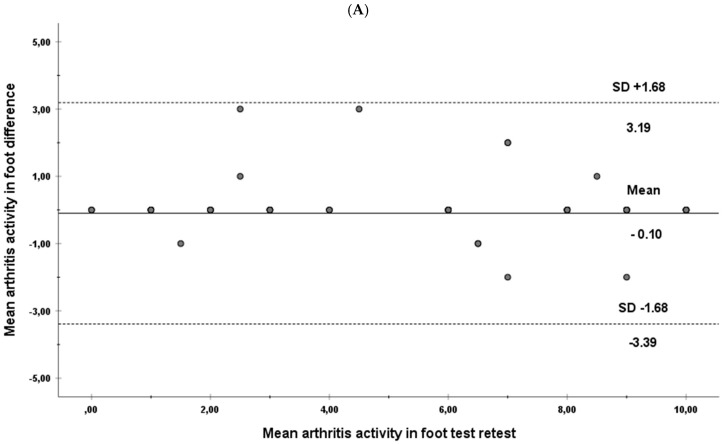

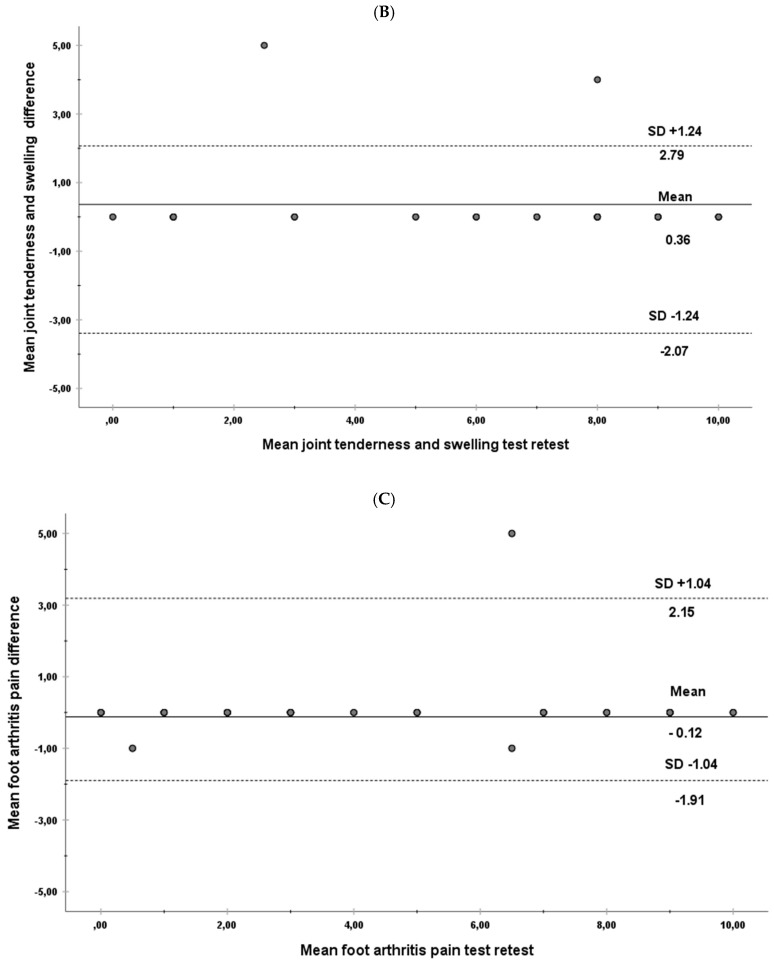

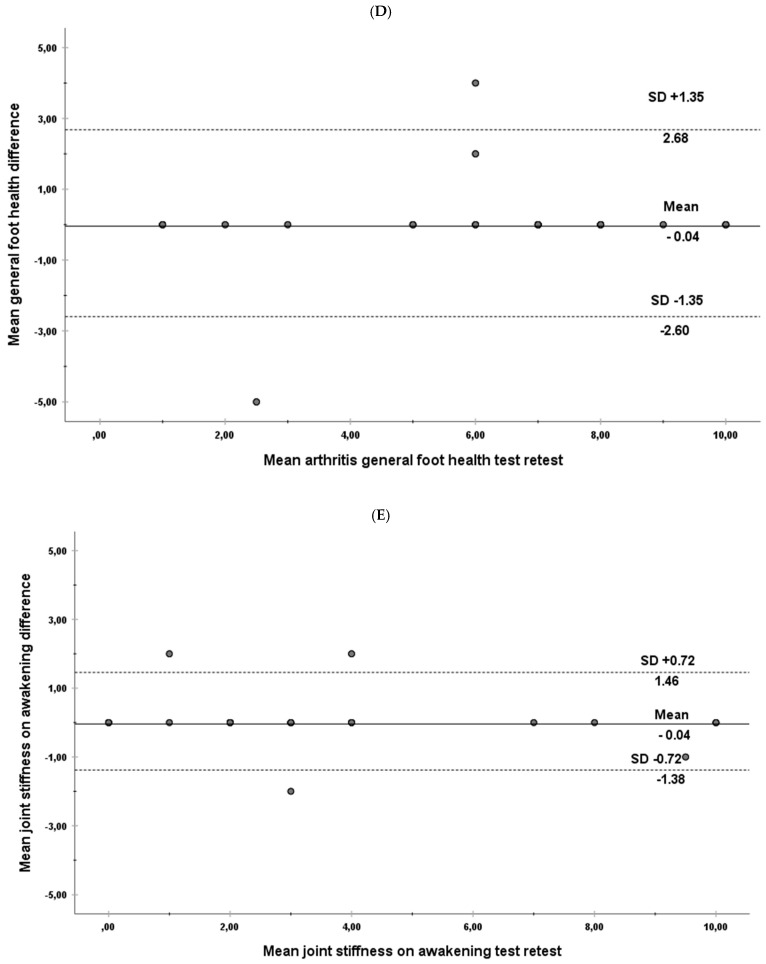

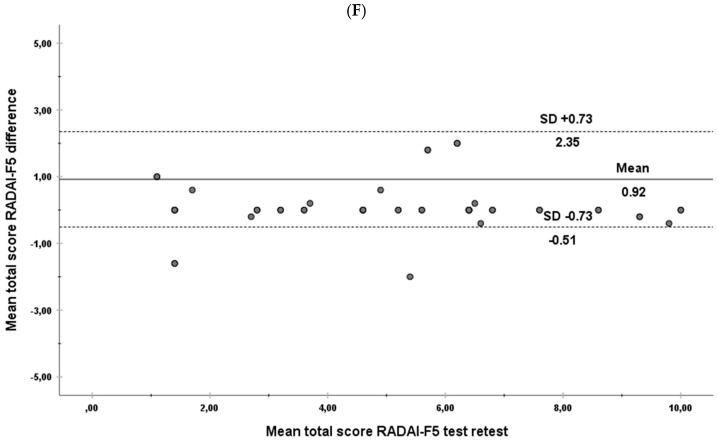
Altman plot showing the agreement between test and retest for the individual subscales and the total score. Dimensions: (**A**) arthritis activity in foot, (**B**) joint tenderness and swelling, (**C**) foot arthritis pain, (**D**)general foot health, (**E**) joint stiffness on awakening sub-scales. (**F**) total score RADAI-F5.

Figure 1A–F show the Bland–Altman graphs for the test-retest of each dimension evaluated, as well as the total for each subject. The calculation of the difference between the means of each session was within the 95% confidence interval in all subjects and dimensions, and gave very similar results.

## 4. Discussion

According to the results obtained in the present investigation, the translated RADAI-F5 questionnaire in Spanish can be used as a valid questionnaire to measure the involvement of rheumatoid arthritis locally in the foot, arthritis activity in foot, joint tenderness and swelling, foot arthritis pain, general foot health and joint stiffness on awakening domains in the Spanish population, with the same properties as the RADAI-F5 questionnaire in English.

As the original FAOS was validated in Sweden with a very high degree of reliability and sensitivity changes after clinical interventions [27,28], the objectives were achieved in our research, and the characteristics of this questionnaire, such as its reliability and speed of completion, make it ideal for clinical use. It must not be forgotten to use this questionnaire as a complement to compound indices of disease activity such as PROMS, or in the case of FIS rehabilitation, the latter with the capacity to distinguish between inflammatory or mechanical-functional diseases of the foot, which is essential in early rheumatoid arthritis [3,20]. This cross-cultural adaptation ensures the conditions of reliability, content validity, internal consistency, internal consistency responsiveness and interpretability that the RADAI-F5 questionnaire already had [3].

Examining the health of the feet in patients with systemic diseases such as type II diabetes mellitus has become a focus of research,. Thus, in the investigation by Domínguez-Muñoz et al. [29], it was observed that the dimensions of general foot health, general health and vigor show a medium level value. These data could be useful to compare the health status of the foot in people with type II diabetes mellitus who attend primary care centers, in the same way as the validated RADAI-F5 questionnaire in people with rheumatoid arthritis. It must be highlighted that Domínguez-Muñoz et al. did not perform a cross-cultural adaptation of previous reliability, which adds importance to the present investigation in terms of reliability [29]. There are other cross-cultural adaptations of questionnaires without reliability tests or assessments of clinimetric characteristics into Spanish [30], but the authors of this research think that both studies are necessary to be able to use the questionnaires in clinical settings and research.

Comparing the results of our research and its success in Spanish cross-cultural validation with other validations carried out in Spanish for assessments of foot health, it can be affirmed that the results of the Spanish version of the F-F-I (F-F-I-Sp) obtained an internal consistency of 0.96 with regard to disability dimension, which was considered as a validated and credible clinimetric result. Hence, our results with a Cronbach’s alpha of 0.980 should be qualified and considered in the same way [31].

Moreover, the Manchester Spanish [MFPDI] questionnaire was also employed with the F-F-I in a population of arthritis patients linked with healthy patients with significant differences, especially with a VAS of 6 to 2 for arthritis patients with foot pain vs. arthritis foot patients without foot pain, in the research of Reinoso-Cobo et al. [32].

In addition, the Medical Outcomes Study 36-item Short-Form Health Survey (SF-36) [33] is a popular tool for assessing health-related quality-of-life and has been used in many physical health conditions and healthcare settings [34]. The SF-36 has been found to be a reliable and valid measure in rheumatoid arthritis [35] and has been used in numerous studies to assess the quality of life of patients with this disease [33]. The psychometric validation of the SF-36 into Yoruba language had positive results. Data were analyzed using Pearson’s product moment correlation analysis, independent *t*-test, one-way analysis of variance, multi-trait scaling analysis and intra-class correlation (ICC) at *p* < 0.05 [36].

Furthermore, the psychometric validation of the SF-36 into Greek has demonstrated construct validity with a Cronbach’s alpha coefficient that met the criterion (>0.70) [37]. The validation of the SF-36 questionnaire into Dutch obtained a result for Cronbach’s alpha coefficient exceeding 0.70 [34]. The results of our study showed results similar to this achievement, due to the fact that the Cronbach’s alpha results were 0.914 and 0.918 pretest and posttest, respectively.

In general, despite its brevity, the SF12 is comparable to the SF36 with only a certain loss of performance in the area of health improvements. Physical function has a highly correlated evaluation between both questionnaires; however the mental health scale is a dimension not so clinically capable of evaluating the SF-12 questionnaire [38], which is a questionnaire also used in rheumatoid arthritis [39]. A study for the validation of the SF-12 in six Asian countries showed adequate psychometric properties, including high reliability (Cronbach’s alpha = 0.85) [40]. In our study, the results can be related to HQol dimensions, as in the case of arthritis pain in the foot, whose Cronbach’s alpha results were 0.953.

Subjects in the original RADAI-F5 questionnaire and validations of the SF-36 questionnaire in rheumatoid arthritis were selected from patients treated by different rheumatology consultation hospitals [3,33], and from an age group between 18 and 75 years.

Furthermore, comparing different cultural contexts, we can say that the results shown by the RADAI-F5 are on the same level as another clinimetric tool regarding foot health disabilities and frailty degree, and for this reason, could be taken into consideration to measure daily living activity domains in future research [41,42].

In our study, the questionnaire respondents were recruited from groups or associations of patients with rheumatoid arthritis and not necessarily in the hospital setting, which could be a possible limitation because the hospital setting assumes a higher level of morbidity of the subjects [33]. Another limitation of the study may be that the test-retest was influenced by a possible treatment such as infiltration. This possible limitation had already been taken into consideration by the authors of RADAI-F5, but it must also be taken into account [3] here.

## 5. Conclusions

The RADAI-F5 es is a useful and trustworthy clinimetric tool that appropriately applies to the Spanish community and can be managed as a whole or in terms of its respective dimensions, such as arthritis activity in the foot, joint tenderness and swelling, and foot arthritis pain sub-scales.

## Figures and Tables

**Table 1:** Sociodemographic characteristics of the sample population.

	Total Group n 50 Mean ± SD Range N = 79	Men n 27 Mean ± SD Range N = 24	Women n 23 Mean ± SD Range N = 55	*p* Value
Age, years	69.220 ± 17.136 (64.349–74.090)	64.037 ± 18.107 (56.873–72.200)	42.018 ± 13.959 (69.267–81.340)	0.029
Weight (kg)	66.400 ± 15.877 (61.887–70.912)	67.537 ± 16.053 (61.186–73.887)	65.065 ± 15.920 (58.180–71.949)	0.533
Height (cm)	1.633 ± 0.088 (1.607–1.658)	1.671 ± 0.066 (1.645–1.697)	1.587 ± 0.091 (1.548–1.627)	0.002
BMI (kg/m^2^)	24.854 ± 5.541 (23.221–24.428)	24.074 ± 5.205 (22.014–26.133)	25.769 ± 5.894 (23.221–28.318)	0.316

*Abbreviations*: BMI, body mass index; SD, standard deviation. In all the analyses, *p* < 0.05 (with a 95% confidence interval) was considered statistically significant. *p*-values are from Mann–Whitney U test.

**Table 2:** Results of test-retest reliability, Item–total correlation and systematic differences of the RADAI-F5 according to each domain.

	Test (N = 50)			Retest (N = 50)			Correlation Test-Retest	Reliability Test-Retest	Systematic Differences Test-Retest
DOMAIN	Mean ± SD (95% CI)	Item–Total Correlation r (*p*) *	Alpha If Item Removed	Mean ± SD (95% CI)	Item–Total Correlation r (*p*) *	Alpha If Item Removed	Item–Total Correlation r (*p*) *	Alpha If Item Removed	Mean ± SD (95% CI)
Arthritis activity in foot	5.540 ± 3.252 (4.61–6.46)	0.865 (<0.01)	0.896	5.64–3.30 (4.70–6.57)	0.839 (<0.01)	0.885	0.858 (<0.01)	0.953 (0.876–0.960)	0.812
Joint tenderness and swelling	6.080 ± 3.306 (5.14–7.019)	0.898 (<0.01)	0.894	5.72 ± 3.41 (4.75–6.68)	0.862 (<0.01)	0.883	0.914 (<0.01)	0.952 (0.938–0.970)	0.063
Foot arthritis pain	4.60 ± 3.41 (3.63–5.56)	0.965 (<0.01)	0.879	4.48 ± 3.26 (3.55–5.40)	0.932 (<0.01)	0.878	0.965 (<0.01)	0.951 (0.948–0.969)	0.914
General foot health	6.08 ± 2.85 (5.26–6.89)	0.763 (<0.01)	0.910	6.04 ± 2.59 (5.30–6.77)	0.671 (<0.01)	0.915	0.865 (<0.01)	0.959 (0.884–0.961)	0.916
Joint stiffness on awakening sub-scales	3.72 ± 3.103 (2.83–4.60)	0.446 (<0.01)	0.946	3.68 ± 3.22 (2.76–4.59)	0.427 (<0.01)	0.948	0.935 (<0.01)	0.965 (0.948–0.970)	0.557
Total	5.20 ± 2.60 (4.46–5.94)	N/A	0.875	5.11 ± 2.57 (4.37–5.84)	N/A	0.871	0.933 (<0.01)	0.951 (0.944–0.988)	0.347
	Total Cronbach alpha test: 0.914			Total Cronbach alpha retest: 0.918					

*Abbreviations:* SD, standard deviation; CI 95%; confidence interval 95%; ICC, intraclass correlation coefficient; N/A, not applicable; * Spearmen test; *p* values < 0.05 are considered significant.

**Table 3:** Results of test-retest reliability, Item–total correlation and systematic differences of the RADAI-F5 according to each item.

	Test (N = 50)		Retest (N = 50)			Correlation Test-Retest	Reliability Test-Retest		Systematic Differences Test-Retest	
						Corrected Item-Total Correlation	Cronbach’s Alpha if Item Deleted	r (*p*) *	ICC (IC95%)	r (*p*) *
Item	Mean ± SD (95% CI)	Corrected Item-Total Correlation	Cronbach’s Alpha if Item Deleted	Mean ± SD (95% CI)	Corrected Item-Total Correlation	Cronbach’s Alpha if Item Deleted	r (*p*) *	ICC (IC95%)	r (*p*) *	0.812
Item 1: THINKING ONLY OF YOUR FEET How active was your arthritis IN YOUR FEET over the last 6 months?	5.54 ± 3.25 (4.61–6.46)	0.865 (<0.01)	0.896	5.64–3.30 (4.70–6.57)	0.839 (<0.01)	0.885	0.858 (<0.01)	0.953 (0.876– 0.960)	0.812	0.063
Item 2: THINKING ONLY OF YOUR FEET How active is your FOOT arthritis today with respect to joint tenderness and swelling?	6.08 ± 3.30 (5.14–7.01)	0.898 (<0.01)	0.894	5.72 ± 3.41 (4.75–6.68)	0.862 (<0.01)	0.883	0.914 (<0.01)	0.952 (0.938–0.970)	0.063	0.914
Item 3: THINKING ONLY OF YOUR FEET How severe is your arthritis pain IN YOUR FEET today?	4.60 ± 3.41 (3.63–5.56)	0.965 (<0.01)	0.879	4.48 ± 3.26 (3.55–5.40)	0.932 (<0.01)	0.878	0.965 (<0.01)	0.951 (0.948–0.969)	0.914	0.916
Item 4: THINKING ONLY OF YOUR FEET How would you describe your general FOOT health today?	6.08 ± 2.85 (5.26–6.89)	0.763(<0.01)	0.910	6.04 ± 2.59 (5.30–6.77)	0.671 (<0.01)	0.915	0.865 (<0.01)	0.959 (0.884–0.961)	0.916	0.557
Item 5: THINKING ONLY OF YOUR FEET Did you experience foot joint stiffness on awakening yesterday morning? If yes, how long was this stiffness IN YOUR FEET?	3.72 ± 3.10 (2.83–4.60)	0.446 (<0.01)	0.946	3.68 ± 3.22 (2.76–4.59)	0.427 (<0.01)	0.948	0.935 (<0.01)	0.965 (0.948–0.970)	0.557	

*Abbreviations*: SD, standard deviation; 95% CI; 95% confidence interval; ICC, intraclass correlation coefficient; N/A, not applicable; * Spearman (rs) test *p* values < 0.05 are considered as statistically significant.

## Data Availability

The dataset supporting the conclusions of this article is available in the corresponding author of this research.
